# Supplementary material for: Evaluation of potential reference genes for real-time qPCR analysis in a biparental beetle, Lethrus apterus (Coleoptera: Geotrupidae)
Source: PeerJ. 2017 Nov 28;5:e4047. doi: 10.7717/peerj.4047 (PMC5710163; doi:10.7717/peerj.4047)
Supplement: Supplementary file 1 [file peerj-05-4047-s001.odt]

| Gene | All samples | Head samples | Thorax samples |
| --- | --- | --- | --- |
|  |  | All head samples | Male head samples | Female head samples | All thorax samples | Male thorax samples | Female thorax samples |
|  | M value | M value | M value | M value | M value | M value | M value |
| GAPDH | 1.531 | 1.302 | 1.428 | 1.184 | 1.648 | 1.538 | 1.769 |
| TUB1a | 1.529 | 1.296 | 1.142 | 1.390 | 1.552 | 1.510 | 1.600 |
| EF1a | 1.267 | 1.488 | 1.647 | 1.122 | 1.047 | 0.652 | 1.352 |
| EF2 | 0.517 | 0.492 | 0.488 | 0.504 | 0.560 | 0.596 | 0.477 |
| ARF1 | 0.906 | 0.853 | 0.885 | 0.824 | 0.959 | 0.941 | 0.982 |
| ARF4 | 1.609 | 1.405 | 1.651 | 1.330 | 1.640 | 1.369 | 1.822 |
| RPS8 | 0.390 | 0.304 | 0.346 | 0.264 | 0.358 | 0.288 | 0.222 |
| RP4 | 0.614 | 0.696 | 0.597 | 0.754 | 0.477 | 0.464 | 0.510 |
| L7A | 0.390 | 0.440 | 0.451 | 0.437 | 0.358 | 0.288 | 0.378 |
| L10 | 1.313 | 1.672 | 1.334 | 1.774 | 1.101 | 0.740 | 1.227 |
| RP18 | 0.419 | 0.304 | 0.346 | 0.264 | 0.439 | 0.504 | 0.222 |
