# Supplementary material for: Evaluation of potential reference genes for real-time qPCR analysis in a biparental beetle, Lethrus apterus (Coleoptera: Geotrupidae)
Source: PeerJ. 2017 Nov 28;5:e4047. doi: 10.7717/peerj.4047 (PMC5710163; doi:10.7717/peerj.4047)
Supplement: Supplementary file 2 [file peerj-05-4047-s002.odt]

| Rank | All samples | Head samples | Thorax samples |
| --- | --- | --- | --- |
|  | Gene | SV | Gene | SV | Gene | SV |
| 1 | L7A | 0.126 | L7A | 0.116 | L7A | 0.096 |
| 2 | RP4 | 0.144 | EF2 | 0.179 | EF2 | 0.114 |
| 3 | RPS8 | 0.215 | RP4 | 0.190 | RP4 | 0.130 |
| 4 | EF2 | 0.221 | RP18 | 0.200 | RP18 | 0.135 |
| 5 | EF1a | 0.227 | RPS8 | 0.234 | RPS8 | 0.146 |
| 6 | ARF1 | 0.236 | ARF1 | 0.283 | EF1a | 0.154 |
| 7 | RP18 | 0.240 | TUB1a | 0.333 | ARF1 | 0.220 |
| 8 | TUB1a | 0.278 | GAPDH | 0.350 | L10 | 0.249 |
| 9 | L10 | 0.310 | EF1a | 0.393 | TUB1a | 0.296 |
| 10 | GAPDH | 0.352 | ARF4 | 0.404 | ARF4 | 0.328 |
| 11 | ARF4 | 0.352 | L10 | 0.409 | GAPDH | 0.341 |
