# Supplementary material for: Evaluation of potential reference genes for real-time qPCR analysis in a biparental beetle, Lethrus apterus (Coleoptera: Geotrupidae)
Source: PeerJ. 2017 Nov 28;5:e4047. doi: 10.7717/peerj.4047 (PMC5710163; doi:10.7717/peerj.4047)
Supplement: Supplementary file 3 [file peerj-05-4047-s003.odt]

| Gene | All samples | Head samples | Thorax samples |
| --- | --- | --- | --- |
|  |  |  | All head samples | Male head samples | Female head samples | All thorax samples | Male thorax samples | Female thorax samples |
|  | MAD | r value | MAD | r value | MAD | r value | MAD | r value | MAD | r value | MAD | r value | MAD | r value |
| GAPDH | 1.99 | 0.902 | 1.47 | 0.886 | 1.00 | 0.782 | 1.70 | 0.934 | 2.33 | 0.901 | 2.45 | 0.926 | 2.12 | 0.873 |
| TUB1a | 1.84 | 0.915 | 1.53 | 0.903 | 0.86 | 0.753 | 1.94 | 0.931 | 2.03 | 0.925 | 2.21 | 0.944 | 1.76 | 0.898 |
| EF1a | 1.67 | 0.929 | 1.48 | 0.872 | 1.34 | 0.902 | 1.57 | 0.933 | 1.79 | 0.963 | 1.98 | 0.987 | 1.52 | 0.906 |
| EF2 | 1.45 | 0.966 | 1.29 | 0.959 | 1.07 | 0.957 | 1.33 | 0.955 | 1.63 | 0.973 | 1.95 | 0.991 | 1.24 | 0.934 |
| ARF1 | 1.36 | 0.900 | 1.09 | 0.848 | 0.88 | 0.633 | 1.12 | 0.904 | 1.55 | 0.918 | 1.71 | 0.972 | 1.36 | 0.776 |
| ARF4 | 2.17 | 0.940 | 1.83 | 0.932 | 1.57 | 0.825 | 2.00 | 0.966 | 2.32 | 0.940 | 2.45 | 0.975 | 2.10 | 0.913 |
| RPS8 | 1.53 | 0.964 | 1.28 | 0.963 | 0.83 | 0.920 | 1.42 | 0.973 | 1.68 | 0.966 | 2.02 | 0.982 | 1.30 | 0.923 |
| RP4 | 1.45 | 0.963 | 1.24 | 0.944 | 0.91 | 0.876 | 1.41 | 0.955 | 1.59 | 0.971 | 1.82 | 0.980 | 1.33 | 0.945 |
| L7A | 1.53 | 0.983 | 1.27 | 0.985 | 0.99 | 0.967 | 1.41 | 0.990 | 1.69 | 0.981 | 1.98 | 0.985 | 1.33 | 0.971 |
| L10 | 1.53 | 0.820 | 1.38 | 0.676 | 1.08 | 0.655 | 1.47 | 0.669 | 1.48 | 0.892 | 1.58 | 0.974 | 1.30 | 0.747 |
| RP18 | 1.41 | 0.964 | 1.17 | 0.958 | 0.90 | 0.890 | 1.28 | 0.973 | 1.58 | 0.968 | 1.82 | 0.980 | 1.28 | 0.940 |
