# Supplementary material for: Evaluation of potential reference genes for real-time qPCR analysis in a biparental beetle, Lethrus apterus (Coleoptera: Geotrupidae)
Source: PeerJ. 2017 Nov 28;5:e4047. doi: 10.7717/peerj.4047 (PMC5710163; doi:10.7717/peerj.4047)
Supplement: Supplementary file 4 [file peerj-05-4047-s004.odt]

| Gene | All samples | Head samples | Thorax samples |
| --- | --- | --- | --- |
|  |  | All head samples | Male head samples | Female head samples | All thorax samples | Male thorax samples | Female thorax samples |
|  | SDavg | SDavg | SDavg | SDavg | SDavg | SDavg | SDavg |
| GAPDH | 1.53 | 1.36 | 1.38 | 1.27 | 1.65 | 1.49 | 1.76 |
| TUB1a | 1.46 | 1.42 | 1.26 | 1.49 | 1.48 | 1.40 | 1.54 |
| EF1a | 1.30 | 1.42 | 1.60 | 1.15 | 1.15 | 0.89 | 1.33 |
| EF2 | 1.01 | 0.98 | 0.92 | 0.99 | 0.99 | 0.84 | 1.07 |
| ARF1 | 1.27 | 1.25 | 1.28 | 1.18 | 1.27 | 1.03 | 1.45 |
| ARF4 | 1.61 | 1.52 | 1.59 | 1.37 | 1.64 | 1.33 | 1.84 |
| RPS8 | 1.00 | 0.96 | 0.93 | 0.92 | 1.01 | 0.88 | 1.08 |
| RP4 | 1.03 | 1.05 | 1.01 | 1.03 | 0.99 | 0.88 | 1.05 |
| L7A | 0.93 | 0.91 | 0.90 | 0.87 | 0.93 | 0.83 | 0.99 |
| L10 | 1.55 | 1.68 | 1.44 | 1.79 | 1.37 | 1.03 | 1.56 |
| RP18 | 0.99 | 0.95 | 0.95 | 0.91 | 0.99 | 0.90 | 1.03 |
