# Supplementary material for: Evaluation of potential reference genes for real-time qPCR analysis in a biparental beetle, Lethrus apterus (Coleoptera: Geotrupidae)
Source: PeerJ. 2017 Nov 28;5:e4047. doi: 10.7717/peerj.4047 (PMC5710163; doi:10.7717/peerj.4047)
Supplement: Supplementary file 5 [file peerj-05-4047-s005.odt]

>KY786283

GTATGACAGTAGCTGAAGTTCATCAAGCTTTAGGCCTAGAAGCTCTAAAGAATAGGACATTTCAAATTTTCAAAACATCTGCAACTAAAGGAGAAGGTCTAGATCAAGCAATGGACTGGCTAGCCAATGCTTTACAAAACAG

>KY786282

TAGTACGGACGGTCAAGTCGCCTGAAAATACCGGAGTGCCCATATTGGTCCTCGCGAACAAACAGGATTTACCGGGGGCGCGCGAACCTCGCGAGTTGGAAAAATTGCTCGGTTTGATCGAACTGGGCGGCAGCGGAACACCGGGAGGCCATTTATGGCACGTACAACCCGCATGCGCCATAACAGGTGACGGTCTAC

>KY786281

AAACCTTTGCGTCTTCCACTACAGGTCATTTTATTATTTTAAATTTTGCAATCAATATAATTTTTTTTTATAGGATGTTTATAAAATTGGTGGCATTGGTACAGTACCAGTTGGTCGAGTTGAAACTGGTGTTTTAAAACCAGGTGTTGTGGTAGTCTTTGCACCTGTTGGTCTTACAACTGAAG

>KY786280

GATGAGAAATCCACATGTCCAGGTGAATCAATCAAATTAATCAAAAAACCCTTCTCATCTTTCTCACGTTGGTCGGGATTCGTAATAAATACTAAATCTTTGTCATCAAGCTCAAAGAACATAGATATGGCACTAAAACACCAAACACAAAATAAATAACAAACCAAAAAAAGAAACCATTTTAAATATTAAGTTTCAAATATGTCAGTTGGCCGTTCTTAAAATCATCAAGCAATATCAGTACAGGTCTGTGATTAAGAACAATCATCATTTAAAATAATGGAAAACTAAATCAAATTTACTGTAAATATCTTACGTTGATTTGATTGTGATACAACGTTCCTGTTCATCTTTTCGTGTGTCTGTAAAACGAGTTTCTCCAGCTTTTGCACCAGCAATGATACCCGCCTTTGATACTAGGGAGTCG

>KY786279

GCCATTCCAGTAAGTTTTCCATTGAGTGATGGGATGACCTTTCCAACGGCTTTTGCAGCTCCAGTAGAAGCTGGAATTATGTTTTGACCTGCGCCACGTCCATCACGCCACAATTTACCAGATGGCCCGTCAACAGTCTTTTGTGTAGCAGTAACAGC

>KY786277

TAGCGACTCAACTGTTCAAGGTTCTTGAGAAGTATCGACCAGAGAGTCAACTTCAAAAGAGGCAACGATTGAAAGCGCTCGCCGAAGCTAAAGCAGCAAAAAAAGAAGAACCCCCATCAAAACGTCCAAACACCATCCGTGCTGGCACGAACACTGTAACTAAACTGATTGAGCAGAAAAAGGCACAACTTGTCGTTATTGCACATGACGTCGATCCAATTGAGG

>KY786278

CGTAGAGCCTCGATAACTTGGGCCTTGAAACGATCACTTGAACGTACACTCATGATTGGTTGACCAATGTGAACACGTGCAACAGTACCTTGTGGTTTACCAAAGGCACCACGCATTCCAGTTTGGAGCCTATAAATAAAAATTATGCATATGAGATGACAAAGAATTACAATTAATCTTCTTTTTTTAATTCCACTGAACATGAATACATGAATGCAGGGGACTGTAAAATAGTTCTATGTCCAAAACAATACAGTTTGATAAAGATTTTACCCACCAATAGTTAGCATTGATTCAAAATTATTTATCTATATTTCTAGCATATTCAACATATAATTTATAATTAAACTTTATCAACAGACTCTTTACTAATATGGATTCATTTACACAATGCACCATGCAACGTTTGTTTAAGGAAACATACAAAATATAAAAGTACATACCTATCAGCTCCAGCACATGA

>KY786275

TAATGGACCACGACGCTGTATGCGCCTTCTGTTGCGCATTTTACCCTTACCAGCACGAAAACGTTGCGACTTGTATACCTGCATAAAAAATGTTTTAGAAATATTATTTTCAAAATAAAATATGTTAAATGGGTTATATTGTGAAATATCAGAGCTTTTGATTGTTAAATCATTAATAATCATAAACACGGACCAAAAGCAAATCATCATAAAATTTAGGAATATAATTGAAATAATAATAAAATTGTTTGCTTTACCTTTTGAATGTCAGCCCAAGCTTTGATCCGCCGTAAGAAAATAACTGCTTGTTTAGTTTTGGTAAGTTCCTGAACTTTATCAGAAACTACTAAAGGCAATTCTGGAACTTGATCAACAACATGTCCCTTGCTCATTACTAAAGCTGGTACG

>KY786276

TTGTAACCACATGAACGCCTACGACCACGTGCTCTTTCAAATTTACGTCCCTTGGAGCGAACAAACGGTTTCGTGTGAGAATGTGGAACTCCAGGAGCGGGACCAAAGTGTTTCACTGCTTCACGAGCATTTCTGCGACCTTGTATCAAAACAGTTTTGTTACCAGTAGGTGAACGTAAAGCTAACT

>KY786274

CATTATGTACGTACGAGAGGAGGCAACGTTAAATACAGGGCGTTACGTCTCGACACTGGAAATTTTGCATGGGGCTCCGAAGGTAGAGCAAAAAGGACGCGTATTATTGATGTAGTTTACAATGCAAGCAACAACGAATTGGTTCGTACCAAAACGTTGGTGAAAAATGCAATCGTCGTTATCGACGCTACTCCCTTTAGA

>KY786273

CAGACTGCACGTTGGACTTTAGCCAAATCACCACCTGGAACAACGGTTGGTGGTTGATAGTTGATGCCAACTTTGAAACCAGTTGGACACCAATCTACGAACTGAATAGTTCTCTTTGTCTTGATGGTCGCTATTGCAGCATTTACATCCTTGGGGACAACATCTCCTCTGTA
