# Supplementary material for: Evaluation of potential reference genes for real-time qPCR analysis in a biparental beetle, Lethrus apterus (Coleoptera: Geotrupidae)
Source: PeerJ. 2017 Nov 28;5:e4047. doi: 10.7717/peerj.4047 (PMC5710163; doi:10.7717/peerj.4047)
Supplement: Supplementary file 6 [file peerj-05-4047-s006.odt]

>GAPDH

GCCATTCCAGTAAGTTTTCCATTGAGTGATGGGATGACCTTTCCAACGGCTTTTGCAGCTCCAGTAGAAGCTGGAATTATGTTTTGACCTGCGCCACGTCCATCACGCCACAATTTACCAGATGGCCCGTCAACAGTCTTTTGTGTAGCAGTAACAGC

>TUB1a

CAGACTGCACGTTGGACTTTAGCCAAATCACCACCTGGAACAACGGTTGGTGGTTGATAGTTGATGCCAACTTTGAAACCAGTTGGACACCAATCTACGAACTGAATAGTTCTCTTTGTCTTGATGGTCGCTATTGCAGCATTTACATCCTTGGGGACAACATCTCCTCTGTA

>EF1a

AAACCTTTGCGTCTTCCACTACAGGATGTTTATAAAATTGGTGGCATTGGTACAGTACCAGTTGGTCGAGTTGAAACTGGTGTTTTAAAACCAGGTGTTGTGGTAGTCTTTGC

ACCTGTTGGTCTTACAACTGAAGA

>EF2

GATGAGAAATCCACATGTCCAGGTGAATCAATCAAATTAATCAAAAAACCCTTCTCATCTTTCTCACGTTGGTCGGGATTCGTAATAAATACTAAATCTTTGTCATCAAGCTCAAAGAACATAGATATGGCAGTTGATTTGATTGTGATACAACGTTCCTGTTCATCTTTTCGTGTGTCTGTAAAACGAGTTTCTCCAGCTTTTGCACCAGCAATGATACCCGCCTTTGATACTAGGGAGT

>ARF1

GTATGACAGTAGCTGAAGTTCATCAAGCTTTAGGCCTAGAAGCTCTAAAGAATAGGACATTTCAAATTTTCAAAACATCTGCAACTAAAGGAGAAGGTCTAGATCAAGCAATGGACTGGCTAGCCAATGCTTTACAAAACAG

>ARF4

TAGTACGGACGGTCAAGTCGCCTGAAAATACCGGAGTGCCCATATTGGTCCTCGCGAACAAACAGGATTTACCGGGGGCGCGCGAACCTCGCGAGTTGGAAAAATTGCTCGGTTTGATCGAACTGGGCGGCAGCGGAACACCGGGAGGCCATTTATGGCACGTACAACCCGCATGCGCCATAACAGGTGACGGTCTAC

>RPS8

CATTATGTACGTACGAGAGGAGGCAACGTTAAATACAGGGCGTTACGTCTCGACACTGGAAATTTTGCATGGGGCTCCGAAGGTAGAGCAAAAAGGACGCGTATTATTGATGTAGTTTACAATGCAAGCAACAACGAATTGGTTCGTACCAAAACGTTGGTGAAAAATGCAATCGTCGTTATCGACGCTACTCCCTTTAGA

>RP4

TAATGGACCACGACGCTGTATGCGCCTTCTGTTGCGCATTTTACCCTTACCAGCACGAAAACGTTGCGACTTGTATACCTTTTGAATGTCAGCCCAAGCTTTGATCCGCCGTAAGAAAATAACTGCTTGTTTAGTTTTGGTAAGTTCCTGAACTTTATCAGAAACTACTAAAGGCAATTCTGGAACTTGATCAACAACATGTCCCTTGCTCATTACTAAAGCTGGTACG

>L7A

TAGCGACTCAACTGTTCAAGGTTCTTGAGAAGTATCGACCAGAGAGTCAACTTCAAAAGAGGCAACGATTGAAAGCGCTCGCCGAAGCTAAAGCAGCAAAAAAAGAAGAACCCCCATCAAAACGTCCAAACACCATCCGTGCTGGCACGAACACTGTAACTAAACTGATTGAGCAGAAAAAGGCACAACTTGTCGTTATTGCACATGACGTCGATCCAATTGAGG

>L10

CGTAGAGCCTCGATAACTTGGGCCTTGAAACGATCACTTGAACGTACACTCATGATTGGTTGACCAATGTGAACACGTGCAACAGTACCTTGTGGTTTACCAAAGGCACCACGCATTCCAGTTTGGAGCCTATCAGCTCCAGCACATGA

>RP18

TTGTAACCACATGAACGCCTACGACCACGTGCTCTTTCAAATTTACGTCCCTTGGAGCGAACAAACGGTTTCGTGTGAGAATGTGGAACTCCAGGAGCGGGACCAAAGTGTTTCACTGCTTCACGAGCATTTCTGCGACCTTGTATCAAAACAGTTTTGTTACCAGTAGGTGAACGTAAAGCTAACT
